# Integrative metabolomic profiling reveals aberrations in myometrium associated with adenomyosis: a pilot study

**DOI:** 10.1186/s12958-022-00914-5

**Published:** 2022-03-09

**Authors:** Wei Song, Zhibo Zhang, Ying Jiang, Yang Cao, Bo Zhang, Yujie Wang, Honghui Shi, Lan Zhu

**Affiliations:** 1 Medical Science Research Center, State Key Laboratory of Complex Severe and Rare Diseases, Peking Union Medical College Hospital, Chinese Academy of Medical Science and Peking Union Medical College, Beijing, 100730 China; 2 Department of Obstetrics and Gynecology, Peking Union Medical College Hospital, Chinese Academy of Medical Sciences and Peking Union Medical College, National Clinical Research Center for Obstetric and Gynecologic Disease, Beijing, 100730 China; 3 Department of Pathology, Peking Union Medical College Hospital, Chinese Academy of Medical Sciences and Peking Union Medical College, Beijing, 100730 China

**Keywords:** Adenomyosis, Metabolomics, Myometrium, Metabolic biomarkers

## Abstract

**Background:**

Uterine adenomyosis is a common gynecologic disease in premenopausal women, the pathological mechanism of which remains largely unknown. The aim of this study was to identify metabolic biomarkers significantly altered in the myometrium of adenomyosis patients.

**Methods:**

The comprehensive metabolomic profiles of 17 myometrium specimens from adenomyosis patients and 25 control specimens were analyzed using untargeted approach by combination of gas chromatography–mass spectrometry and high performance liquid chromatography-mass spectrometry. Metabolic data were filtered using orthogonal partial least square-discriminant analysis and univariate statistics.

**Results:**

We firstly demonstrated that the myometrial metabolome of women with adenomyosis is distinct from that of women without adenomyosis. A total of 106 metabolites, mainly including nucleosides, lipids (including acylcarnitines), amino acids, organic acids and carbohydrates, were found to be differentially expressed in myometrium of uteri with adenomyosis compared to the control subjects. Functional inferences of these perturbed metabolites indicated that inflammation, oxidative stress, cell proliferation and apoptosis, and energy metabolism appeared to be involved in the progress of adenomyosis.

**Conclusion:**

This study firstly described the integrated metabolic signatures of the adenomyosis uterus, which provided novel insights for the pathogenesis study of this disease.

**Supplementary Information:**

The online version contains supplementary material available at 10.1186/s12958-022-00914-5.

## Background

Adenomyosis is a benign uterine disease caused by the presence of endometrial glands and interstitial invasion within the myometrium, which was one of the most common disease in women of reproductive age [1]. Symptoms arose by adenomyosis, such as abnormal uterine bleeding, dysmenorrhea, chronic pelvic pain, and infertility, have severe effect on the life quality of these women [2, 3]. So far there is no effective therapy in clinic except for managing symptoms [4]. Although adenomyosis has been studied extensively and various hypotheses have been put forward to explain the pathogenesis, the biological mechanism of adenomyosis has not been thoroughly ascertained to date [5]. It has been suggested that there may be more than one mechanism for the pathogenesis of this uterine disorder [6].

As the end product of DNA expression, metabolites can directly reflect the physiology and pathophysiology of many biological samples. Consistently, metabolomics has developed as a powerful tool for understanding metabolic changes, particularly those small molecules (< 1000 Da), in response to pathophysiological conditions [7]. Metabolomics approach based on nuclear magnetic resonance spectroscopy (NMR) or chromatography coupled with mass spectrometry (MS) could quantify a large number of molecules efficiently from single biological sample, and thus identify metabolites and pathways affected by diseases [8]. From technical aspect, gas chromatography (GC) or liquid chromatography (LC) coupled with MS was considered to be more sensitive and displayed higher-throughput compared to the NMR method [9], which provides new opportunities to better understand the pathophysiology of adenomyosis.

However, few studies have used metabolomics approach to elucidate the global metabolic changes related to adenomyosis, let alone how metabolic pathways are affected in this disease [10, 11]. According to the only study published to date, serum metabolic profiles were changed in women with adenomyosis when compared to the controls, though metabolites of a small subset were picked out [12]. Given the inherent limited resolution of proton nuclear magnetic resonance (^1^H-NMR)-based platform and perhaps the wide heterogeneity of serum subjects, the metabolic framework that supports adenomyosis development deserves further exploration.

In this pilot study, we were interested in changes in the myometrial metabolome of adenomyosis patients. Using the integrated GC–MS and LC–MS based untargeted metabolomics approach, we conducted a cross-sectional study in order to gain a global metabolic insights into the pathological mechanism of adenomyosis. As a result, 106 significant altered metabolites related to oxidative stress, inflammation, cell proliferation, and energy homeostasis were obtained from the myometrium subjects of women with and without adenomyosis, most of which were firstly reported for adenomyosis.

## Materials and methods

### Participants and sample collection

The study was approved by the Ethics Committee of Peking Union Medical College Hospital (No. ZS-2025). We recruited 41 women who undergoing hysterectomy in Peking Union Medical College Hospital between July 2019 and January 2020. For the adenomyosis group, lesions-surrounding myometrial tissue samples were obtained from 17 adenomyosis patients. For the control group, normal myometrial tissue samples were collected from patients without adenomyosis undergoing hysterectomy for uterine leiomyomas (*n* = 16) or cervical intraepithelial neoplasias III (*n* = 9). None of the participants received oral contraception or GnRH agonists and all participants reported spontaneous menses in the 3 months prior to surgery. Patients with endometriosis and uterine malignant tumors were excluded from this study. The preoperative diagnosis of adenomyosis was suggested by characteristic clinical manifestations such as heavy menstrual bleeding and dysmenorrhea with uniformly enlarged uterus, and clinical diagnosis was made by magnetic resonance imaging (MRI). The MRI diagnostic criteria followed previous reports [13, 14]. Then definitive diagnosis of adenomyosis was made on histologic analysis following hysterectomy, which was defined by the presence of endometrial glands and stroma glands > 2.5 mm below the endometrial-myometrial interface [15]. The exclusion of adenomyosis in the control group were based on gynecological examination, transvaginal ultrasonography, pelvic MRI, and surgical examination. The time for operation was depended on the patient’s compliance. As shown in Table 1, there were 19 patients (7 from adenomyosis group, 12 from control group) undergoing hysterectomy during the follicular phase, and 18 patients (8 from adenomyosis group, 10 from control group) undergoing hysterectomy during the luteal phase, respectively. The menstrual phases of the other 5 women were indistinct. No significant differences were found in age, body mass index, gravidity, or parity between the two groups (*p* > 0.05).

**Table 1:** Clinical characteristics of patients recruited in this study

Characteristic	Adenomyosis group	Control group	*P* value
	(*n* = 17)	(*n* = 25)	
Age (year)	44.18 ± 3.66	42.36 ± 4.25	0.16
BMI (kg/m^2^)	22.25 ± 2.61	22.8 ± 2.82	0.53
Gravidity	2.06 ± 0.83	1.92 ± 0.95	0.63
Parity	1.23 ± 0.44	1.36 ± 0.50	0.40
Menstrual phase			
Follicular phase	7 (41%)	10 (40%)	
Luteal phase	6 (35%)	12 (48%)	
Unknown	4 (24%)	3 (12%)	

Value was expressed as mean ± standard deviation or number (percentage). *P* value was from Student’s *t*-test

The hysterectomy and sample collection were performed by a senior gynecologist with extensive experience in adenomyosis. Gross pathological examination was conducted during operation and specimens were send for pathological examination after operation (Fig. S1 in additional file). To collect samples for metabolomic analysis, tissue fragments containing only myometrium were sliced in thickness of about 5 mm. Visual inspection was then conducted to exclude minor lesions before they were frozen at − 80 °C until use.

### Workflow of metabolomic analysis

**Fig. 1:**
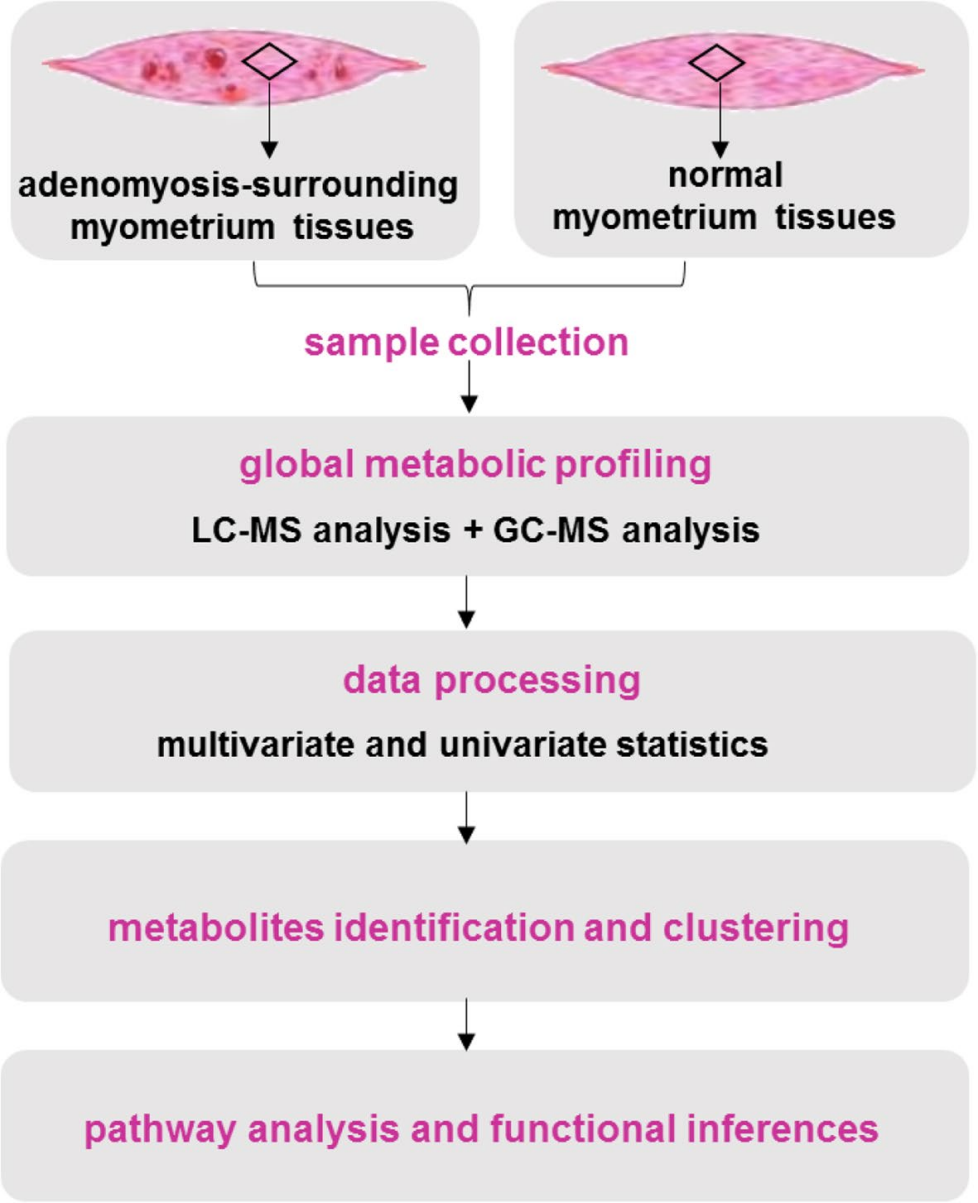
The workflow of this study

The workflow of this study was illustrated in Fig. 1. To obtain global metabolic profiling of the collected samples and detect metabolites as a comprehensive view, metabolomic analysis was conducted using two independent platforms: gas chromatography coupled with mass spectrometry (GC–MS) and ultra-high performance liquid chromatography coupled with mass spectrometry (UHPLC-MS). Dataset from the two platform were used for downstream processing. The discriminative variables were screened out using multivariate and univariate analysis. Significantly altered metabolites were structural characterized and employed for pathway analysis.

### Metabolites extraction

For GC–MS analysis, 75 mg of each sample was extracted with 1500 μL of extraction solution containing acetonitrile, methanol, water (2:2:1, *v*/*v*/*v*), and adonitol (0.5 mg/mL, stock) as internal standard. Samples were vortexed for 30 s and homogenized in ball mill for 4 min, followed by ultrasonication in ice water for 5 min, and incubated at -40 °C for 1 h to precipitate proteins. After centrifugation at 4 °C and 12,000 rpm for 15 min, 300 μL of the supernatant was transferred to a fresh tube. To prepare the quality control (QC) sample, 60 μL of each sample was mixed together. After evaporation in vacuum, 30 μL of methoxyamination hydrochloride (20 mg/mL in pyridine) was added before incubation at 80 °C for 30 min, and then derivatized with 40 μL of BSTFA regent (1% TMCS, *v*/*v*) at 70 °C for 1.5 h. After samples were cooling to room temperature, 5 μL of FAMEs was added to the QC sample.

For UHPLC-MS analysis, 75 mg of the sample were added to 1500 μL of the extract solution (acetonitrile: methanol: water = 2:2:1, *v*/*v*/*v*) containing isotopically-labelled internal standard. After vortex for 30 s, samples were homogenized for 4 min and sonicated for 5 min under ice bath. The procedure above was repeated for 3 times. Then samples were incubated at -40 °C for 1 h and centrifuged at 12,000 rpm and 4 °C for 15 min. Then 400 μL of the supernatant was transferred to a fresh tube and dried in vacuum at 37 °C. The dried samples were reconstituted in 200 μL of 50% acetonitrile by sonication for 10 min. After centrifugation at 13,000 rpm and 4 °C for 15 min, the supernatant was injected to the UHPLC/MS system. QC sample was prepared by mixing an equal aliquot of all samples.

### GC–MS detection

GC–MS analysis was performed on an Agilent 7890 gas chromatograph which was coupled with Agilent 5975C time-of-flight (TOF) mass spectrometer (Agilent Technologies). Sample was separated on a DB-5MS capillary column (30 m × 250 μm × 0.25 μm, J&W Scientific). An aliquot of 1-μL sample was injected into the column under splitless mode while the injector temperature was 280 °C. The column temperature was kept at 50 °C for 1 min, then raised to 310 °C at rate of 10 °C·min^−1^ before it was kept at 310 °C for 8 min. The mass spectrometry data were acquired in full-scan mode with collision energy of -70 eV and m/z ranged from 50 to 500. The scan rate was 12.5 spectra per second and the solvent delay was 6.25 min. The transfer line and ion source temperatures were 280 and 250 °C, respectively. Helium was used as the carrier gas with front inlet purge flow at 3 mL·min^−1^.

### UHPLC-MS detection

UHPLC-MS analysis was performed on an Agilent 1290 UHPLC system (Agilent Technologies) which was equipped with TripleTOF 6600 mass spectrometry (AB Sciex). Sample was separated on a UPLC BEH Amide column (2.1 × 100 mm, 1.7 μm, Waters) with column temperature at 25 °C. The injection volume was 2 μL for each sample. Mobile phase A was acetonitrile. Mobile phase B consisted of ammonium acetate and ammonia hydroxide in water (25 mmol/L, respectively, pH = 9.75). Gradient elution was applied (0–0.5 min, 95% A; 0.5–7.0 min, 95%-65% A; 7.0–8.0 min, 65%-40% A; 8.0–9.0 min, 40% A; 9.0–9.1 min, 40%-95% A; 9.1–12.0 min, 95% A). The mass spectrometry was in tandem with UHPLC via an electrospray ion (ESI) source to acquire MS and MS/MS spectra under IDA mode. In this mode, the top 12 precursor ions from each MS scan (m/z 60–1200) were chosen for MS/MS scan (m/z 25–1200) at collision energy of 30 eV. The cycle time was 0.56 s. Gas 1, gas 2, and curtain gas of the ESI sourse was 60, 60, and 35 psi, respectively. The source temperature was 600 °C. The ion spray voltage was 5000 V and -4000 V in positive and negative ion modes, respectively.

### Data preprocessing and peak annotation

Peak extraction, baseline adjustment, deconvolution, alignment and integration of raw data from GC–MS analysis were performed on Chroma TOF software (version 4.3x, LECO). LECO-Fiehn Rtx5 database was used for metabolites identification by matching their mass spectrum and index of retention time. Peaks detected in less than half of the QC samples or RSD > 30% in QC samples was removed [16]. Raw data files from UHPLC-MS analysis were converted to mzXML format by ProteoWizard (http://proteowizard.sourceforge.net/downloads.shtml), and processed by XCMS software (version 3.2) [17]. This process included peak deconvolution, alignment and integration [18]. An in-house LC–MS/MS database was applied for metabolites identification. The accurate m/z was matched with data from the database, which initially indicated the possible metabolite. Then the putative identification was validated with ion fragments, parent ions and retention time. The concentration of metabolites was determined with the area of the peaks.

### Statistical analysis

A dataset consisted of sample names, peak numbers, and normalized peak areas was imported to SIMCA 15.0 software (Umetrics, Sweden) for multivariate analysis. Data was logarithmic transformed to minimize impact of the high variance of the variables the noise. After this, unsupervised principal component analysis (PCA) was firstly conducted to visualize the distribution of all samples and examine the consistency of QC samples. Secondly, supervised orthogonal partial least square-discriminant analysis (OPLS-DA) was carried out to discriminate the metabolomes and find out significantly altered metabolites among two groups. R^2^ and Q^2^ of the OPLS-DA model were calculated by a seven-fold cross validation, which indicated the goodness-of-fit and the predictive ability of the model, respectively. The overftting of the model was accessed by 200-times permutation test. Furthermore, the variable importance in the projection (VIP) value was used to evaluate the contribution of each variable to the OPLS-DA model. Metabolites with VIP > 1 and *p* < 0.05 (from student *t*-test) were considered as significantly altered metabolites in this study. Adjusted *p*-value (*q*-value) was further determined by the Benjamini–Hochberg false discovery rate (FDR) method [19].

### Pathway analysis

Metabolic pathway analysis was performed on MetaboAnalyst (http://www.metaboanalyst.ca/), a website tool which integrated the KEGG metabolic pathway database (http://www.genome.jp/kegg/) as backend. The significantly affected pathways were screened according to the *p* values of pathway enrichment analysis and the impact values of pathway topology analysis, while impact value > 0.1 and -ln (*p*) > 2.0 were taken as thresholds here [20].

## Results

### Metabolic profiling

**Fig. 2:**
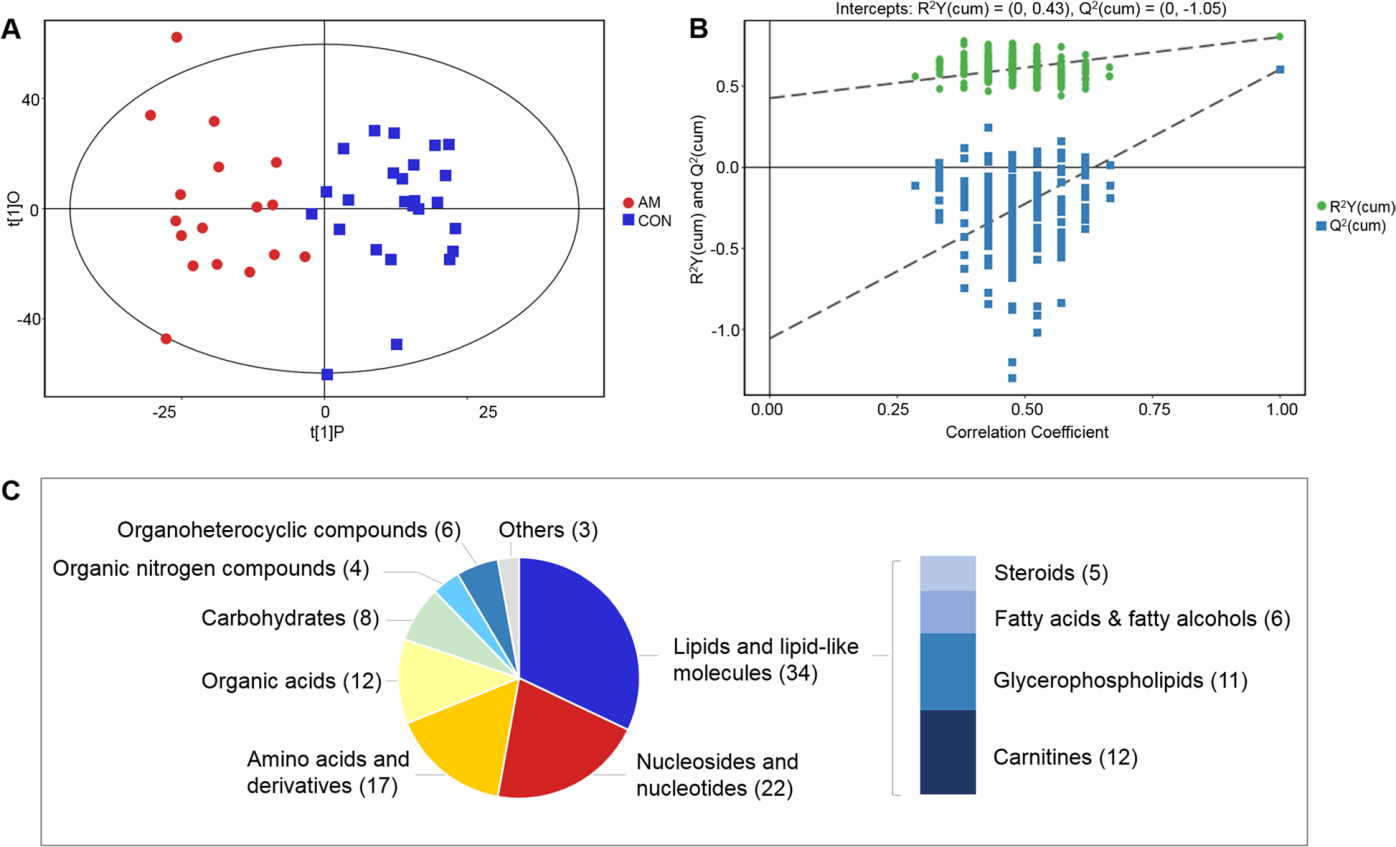
Overview of the metabolomic data. PLS-DA score plots (**A**) with corresponding permutation test plots (**B**) from GC–MS and LC–MS metabolic profiles. The proportion of metabolites’ chemical species which were significantly different between the adenomyosis and control groups (**C**). The number in the brackets represents the amount of corresponding metabolite class

The final dataset containing information of peak number, sample name and peak area was employed for multivariate analysis. To identify dysregulated metabolites related to adenomyosis, we compared all the myometrial tissue specimens from adenomyosis patients against the normal myometrial tissue specimens. Orthogonal partial least-squares discriminant analysis (OPLS-DA) was conducted to visualize the distribution and the grouping of each sample. Clear separations between adenomyosis group and control group was obtained from the score plot of OPLS-DA model with acceptable abilities for reliability and prediction (R^2^Y = 0.802, Q^2^ = 0.603) (Fig. 2A). The Q^2^ from 200-time permutation tests were -1.05, suggesting no overfitting of the OPLS-DA models (Fig. 2B).

**Fig. 3:**
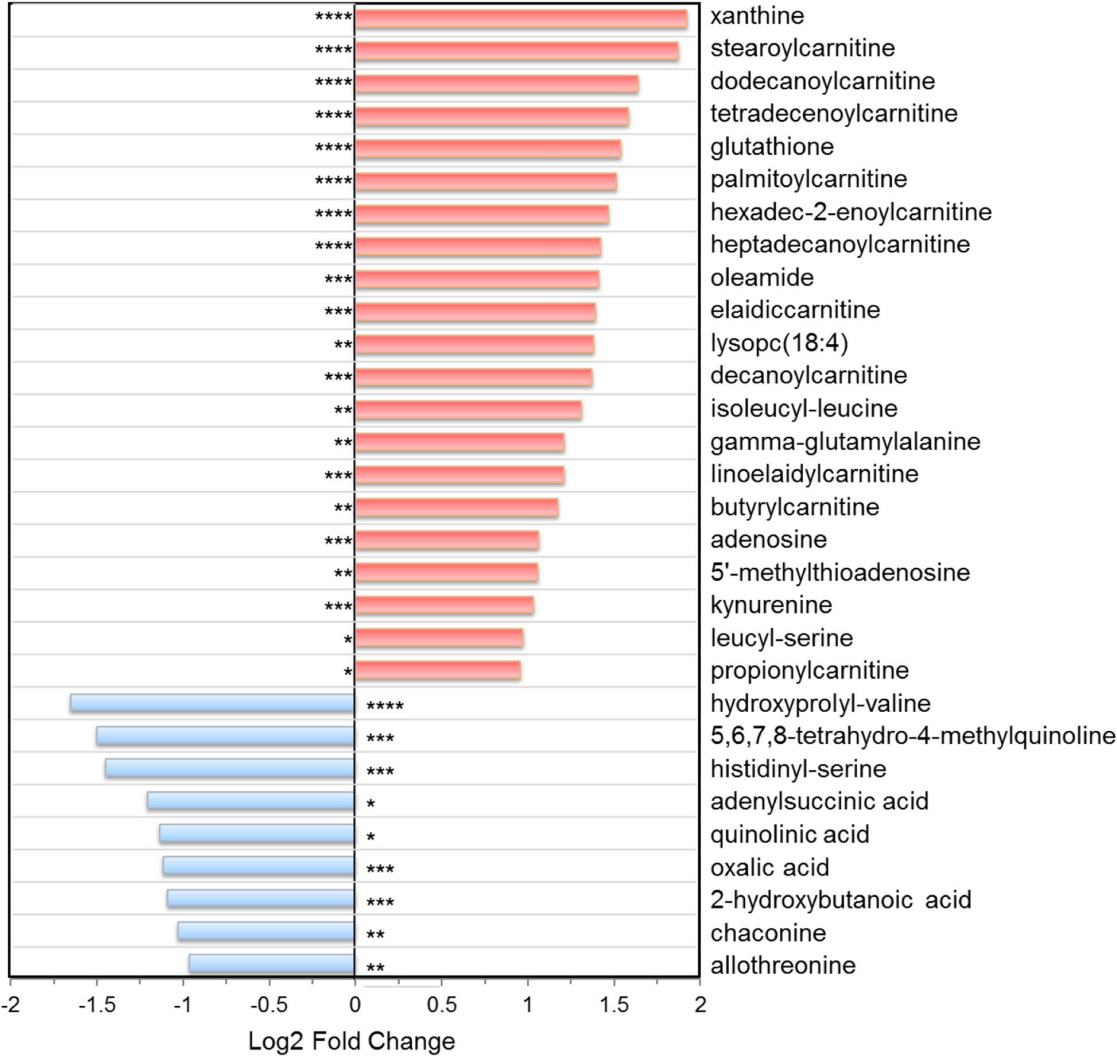
The top 30 differed metabolites between the adenomyosis group and control group. ^****^, *p* < 0.001 and *q* < 0.01; ^***^, *p* < 0.01 and *q* < 0.05; ^**^, *p* < 0.05 and *q* < 0.1; ^*^, *p* < 0.05 and 0.1 < q < 0.15

Based on the variable importance in the projection (VIP) analysis and student’s *t*-test, a total of 106 differential metabolites (79 from LC–MS platform and 27 from GC–MS platform) were identified between the adenomyosis and control groups with VIP > 1.00 and *p* < 0.05 (Table S1 in additional file). Among them, there were 70 metabolites with *q* < 0.1 and 36 metabolites with 0.10 ≤ *q* ≤ 0.15. There are lipids and lipid-like molecules (*n* = 34, 32.1%), nucleosides and nucleotides (*n* = 22, 20.8%), amino acids and analogues (*n* = 17, 16.0%), organic acids (*n* = 12, 11.3%), carbohydrates (*n* = 8, 7.5%), organic nitrogen compounds (*n* = 4, 3.8%), organoheterocyclic compounds (*n* = 3, 2.8%), and metabolites of other categories (*n* = 4, 3.8%) (Fig. 2C). Compared to control group, 76 metabolites were up-regulated in adenomyosis group, while the others were depressed. The top 30 significant metabolites were shown in Fig. 3. To systematically evaluate the perturbed metabolism underlying adenomyosis development, we performed pathway analyses. As a result, we found significant enrichments of purine metabolism, taurine and hypotaurine metabolism, glycerophospholipid metabolism, and nicotinate and nicotinamide metabolism between the AM and control groups (Table 2, Fig. S2 in additional file).

**Table 2:** Significantly dysregulated pathways predicted by pathway enrichment analysis

Pathway	Raw *p*	Impact	Hit metabolites
Purine metabolism	0.001	0.159	xanthine, phosphoribosyl formamidocarboxamide, adenylsuccinic acid, adenosine, inosine, adenine, hypoxanthine, guanine, 5-aminoimidazole ribonucleotide
Taurine and hypotaurine metabolism	0.020	0.353	taurine, 2-hydroxyethanesulfonate, pyruvic acid
Glycerophospholipid metabolism	0.026	0.204	citicoline, O-phosphoethanolamine, glycerylphosphorylethanolamine, CDP-ethanolamine
Nicotinate and nicotinamide metabolism	0.039	0.105	quinolinic acid, maleamate, NAD, pyruvic acid
Cysteine and methionine metabolism	0.081	0.172	5’-methylthioadenosine, S-adenosylhomocysteine, glutathione, pyruvic acid

### Nucleotide metabolism

**Fig. 4:**
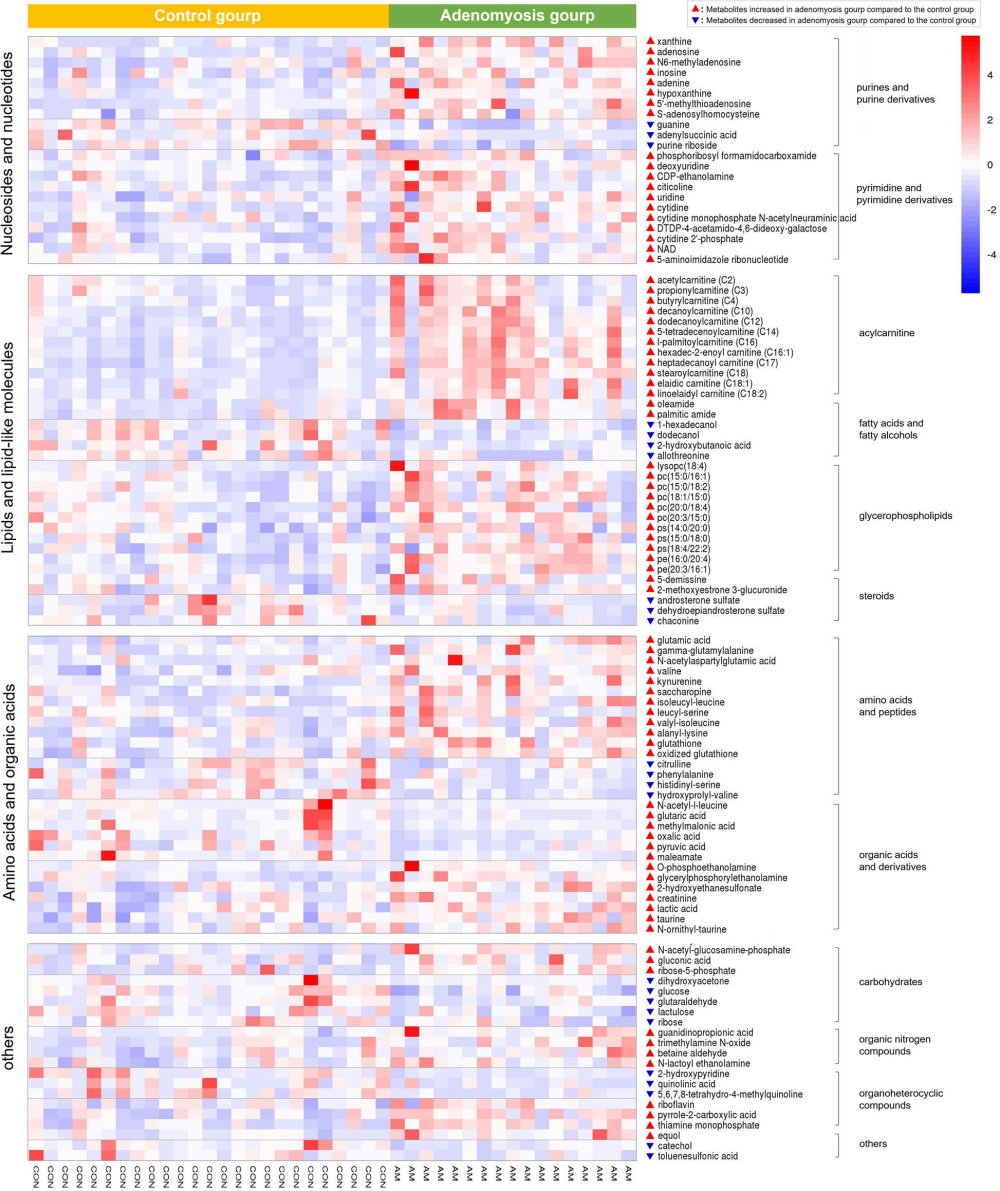
Heat map of the significant altered metabolites between adenomyosis group and control group

Metabolism of nucleotides was perturbed in adenomyosis-surrounding myometrial subjects. As shown in Fig. 3, the most significant metabolite was xanthine (FC = 3.79, *q* = 0.006), an intermediate product of purine metabolism that up-regulated in adenomyosis group. Guanine (FC = 0.56, *q* = 0.004) and purine riboside (FC = 0.73, *q* = 0.008), the precursor metabolites of xanthine, were notably decreased. Other purine derivative (including hypoxanthine, inosine, adenosine, N6-methyladenosine, adenine, 5’-methylthioadenosine, and S-adenosylhomocysteine), as well as pyrimidine nucleosides (such as uridine, deoxyuridine, cytidine, citicoline, and cdp-ethanolamine), were all found to be increased in the study group. The abundance of each metabolite in individual participants were presented in heat map shown in Fig. 4.

### Acylcarnitines and lipids metabolism

In this study, a multitude of acylcarnitines, including short-chain (C2, C3, and C4), medium-chain (C-10, C-12, and C-14), and long-chain acylcarnitines (C16, C16:1, C17, C18, C18:1, and C18:2), were significantly elevated 1.6- to 3.7-fold in adenomyosis group. Eleven out of the 12 aclycarnitines were included in the top 30 different metabolites between the two groups (Fig. 3). Similar observations were also made for oleamide (FC = 2.66, *q* = 0.023) and palmitic amide (FC = 1.82, *q* = 0.062). Meanwhile, we observed decreased levels of a few fatty acids and fatty alcohols (such as 2-hydroxybutanoic acid, hexadecanol, and dodecanol) in adenomyosis group (Fig. 4).

Glycerophospholipid metabolism was also altered between the study and control groups. Multiple glycerophospholipid subclasses, mainly including phosphatidylcholine (PC), phosphatidylethanolamine (PE), and phosphatidylserine (PS), increased significantly in the adenomyosis group (Fig. 4). In addition, four intermediates in glycerophospholipid metabolism pathway, including citicoline, CDP-ethanolamine, O-phosphoethanolamine, and glycerylphosphorylethanolamine, were also found to be higher in adenomyosis group compared to the controls (Table 2).

Significantly, the levels of specific steroid metabolites within estrogenic and androgenic metabolism differed in AM cases. 2-methoxyestrone 3-glucuronide (metabolite of estrone *in-vivo*) and equol (exogenous estrogen from food sources) were up-regulated (FC = 1.25 and 1.87, *q* = 0.14 and 0.08, respectively), while two main androgen, androsterone and dehydroepiandrosterone, were down-regulated (FC = 0.57 and 0.67, *q* = 0.13 and 0.14, respectively).

### Metabolism of amino acids, organic acids, and carbohydrates

Our metabolomic data showed perturbations of various amino acids and their intermediate products. A number of amino acids, including beta-glutamic acid, gamma-glutamylalanine, N-acetylaspartylglutamic acid, valine, kynurenine, and saccharopine, as well as some dipeptides (e.g., isoleucyl-leucine, leucyl-serine, valyl-isoleucine, and alanyl-lysine) were significantly elevated in myometrium specimens from adenomyosis group. Particularly, glutathione (FC = 2.91, *q* = 0.009), a tripeptide known for its antioxidant and detoxifying properties, as well as oxidized glutathione (FC = 1.53, *q* = 0.014), were also increased in adenomyosis group. In contrast, citrulline, phenylalanine, N-acetyl-leucine, and two dipeptides (histidinyl-serine and hydroxyprolyl-valine) maintained a decreasing pattern in adenomyosis group.

Although taurine does not participate in protein synthesis, it is closely related to the metabolism of cystine and cysteine. In this study, taurine (FC = 1.43, *q* = 0.048) and its two relevant metabolites (N-ornithyl-taurine and 2-hydroxyethanesulfonate) showed upward trends in adenomyosis group. The same patterns were also applied to lactic acid and creatinine, two metabolic products released by the muscle tissues. Besides, the decreased level of oxalic acid, pyruvic acid, glutaric acid, and a number of carbohydrates (glucose, glutaraldehyde, and lactulose, etc.) indicated the disturbances of energy metabolism in the myometrium of adenomyosis patients.

### Metabolism of organic nitrogen compounds and others

Among these differentially abundant metabolites, trimethylamine N-oxide (TMAO), which came from the oxidation of trimethylamine (TMA) by gut microbiota, was significantly elevated in adenomyosis group (FC = 1.61, *q* = 0.097). Moreover, some precursors of TMAO, including betaine aldehyde, creatinine, and acetylcarnitine (the last two have been mentioned above), were all showed the same trends. We also observed some perturbations in metabolism of nicotinate and nicotinamide, with decreased levels of maleamate, quinolinic acid, and pyruvic acid, as well as increased level of NAD in adenomyosis group.

## Discussion

In this pilot study, the metabolic phenotype and the underlying metabolic pathways involved in the myometrium of adenomyosis patients were first elucidated using nontargeted GC–MS and LC–MS platforms. Instead of serum, urine, or other body fluid, myometrium around the lesion may contain more information about the presence of adenomyosis and give more direct evidence in the pathological process. Therefore the myometrium tissues were engaged and several interesting findings arose from our data.

Carnitine plays a pivotal role in in transporting fatty acids across the mitochondrial membrane for β-oxidation. As intermediates of carnitine metabolism, acylcarnitines could reflect the mitochondrial and oxidative stress. Its abnormal accumulation points also towards the mitochondrial dysfuction and inflammatory state, which have been proved to be associated with adenomyosis [21, 22]. A previous study has reported that the plasma levels of acylcarnitines were significantly elevated in endometriosis patients, and a panel of these metabolites showed potential as diagnostic biomarkers of endometriosis [23]. Our study demonstrated, for the first time, significant increase of a dozen of acylcarnitines in myometrium tissues from adenomyosis patients.

Except for disturbed mitochondrial stress, elevated levels of these acylcarnitines also suggested activated fatty acids oxidation and perturbed energy metabolism. Consequently, decreased levels of some fatty acids and fatty alcohols (such as 2-hydroxybutanoic acid, 1-hexadecanol, dodecanol, and allothreonine) were observed in myometrium derived from women with adenomyosis when compared to the controls. Previous studies have revealed that the decreased level of fatty acids was involved in the mechanism of fibrogenesis, which could impair the architecture and organ function of normal tissue [24, 25]. Fibrosis is also a pathological feature of adenomyosis that can be secondary to the infiltration of endometrium into the myometrium [26–28]. Ectopic endometrial tissue induces smooth muscle cell hypertrophy and hyperplasia that are a reflection of a reaction of the surrounding tissue [28]. Several studies have demonstrated the extensive distribution of fibrosis in the myometrium of uteri with adenomyosis [29, 30]. During sample collection in this study, we also found that samples taken from the surrounding myometrium of ectopic endometrium showed higher stiffiness than those from the uteri without adenomyosis, which was in consistent with the fibrotic characteristics [31]. Therefore the decreased level of fatty acids in adenomyosis group was probably associated with the fibrogenesis after myometrium injury.

Another interesting finding of this study is that the myometrium level of TMAO, a gut microbiota-derived metabolite and main intermediate of choline metabolism, was significantly increased in adenomyosis patients. It has been report that food-derived PC and carnitines could generate trimethylamine (TMA) during intestinal metabolism, which subsequently produces TMAO [32]. In the present study, Betaine aldehyde, another primary precursor of TMA and TMAO, were also significantly elevated in adenomyosis group. In addition, the serum level of choline, a metabolite can be converted into TMA via choline TMA lyases, was found to be significantly increased in women with adenomyosis, which corroborated with our work to some extent [12]. The elevated TMAO level was strongly associated with increased systematic inflammatory response [33–35], which have been proved to play a critical role in the onset and progression of adenomyosis [5]. Although currently it is difficult to interpret how these microbiome-associated metabolites accumulated in the myometrium of adenomyosis women, these findings could still provide new hypothesis of disease origin for adenomyosis. It might be worthwhile investigating whether gut microbiota was involved in the onset and progression of adenomyosis in the future.

Glycerophospholipids are crucial for cellular membrane integrity and energy storage. Myometrium subjects from adenomyosis group displayed elevated levels in a wide array of glycerophospholipid classes, including PC, PS, and PE. PC is derived primarily from choline and reported to be crutial for both cell proliferative growth and programmed death. The up-regulation of PC synthesis was frequently observed in cancer cells [36], as well as in the eutopic endometrium of endometriosis patients [37]. PC itself is reported to be closely related to inflammation process [22]. Beisdes, evidences have suggested that deregulation of autophagy are closely associated with adenomyosis [5]. PS and PE, two kinds of glycerophospholipids play important role in cellular apoptosis and autophagy, respectively [38, 39], were also notably increased in adenomyosis group. All these increases in lipid metabolites presumably contribute to the development of adenomyosis in multifaceted ways, not only by providing energy source and changing membrane biogenesis in cell proliferation, but also by regulating inflammatory response and cell apoptosis.

Dysregulated amino acid degradation and homeostasis have been implicated as cause of abnormal energy metabolism and cell proliferation in adenomyosis [10, 11]. Our metabolomic data showed perturbations of various amino acids, such as glutamate (glutamic acid), valine, and some peptide derivatives comprising of aspartic acid, alanine, and leucine. Glutamate, alanine and aspartic acid were abundant free amino acids which were associated with energy metabolism as key fuel [40]. In particular, previous studies have proved that glutathione synthesis, which was deeply involved in promoting cell proliferation [41, 42], was activated at the occurrence of adenomyosis. [43]. Our metabolomic data further supported this finding with elevated levels of glutathione and oxidized glutathione in adenomyosis group. Besides, taurine, one of the sulphur-containing amino-acids, has been suggested to work as important antioxidant to protect cells from oxidative stress [44]. This could explain the observed higher levels of taurine and N-ornithyl-taurine in myometrium undergoing oxidative process in adenomyosis group. These results were also proved by previous study, in which increased level of taurine was also observed in urines of endometriosis patients.

Recent study particularly revealed impaired glucose metabolism involved in adenomyosis patients [12]. The present study showed that in the myometrium of adenomyosis patients there was decreased levels of glucose and pyruvic acid, whereas increased levels of lactic acid and creatinine, which were probably derived from the unbalanced conversion of glucose to lactate, as well as the perturbed glycolysis metabolism. In accordance with our study, the serum level of creatinine was found to be increased in the serum of adenomyosis patients [12]. Besides, earlier report also revealed the decreased level of glucose and higher level of lactic acid in cases of endometriosis [45]. These findings also suggested that there were some common metabolic patterns in the onset of adenomyosis and endometriosis, two benign gynecological diseases with similar clinical features. Furthermore, the decreased levels of other carbohydrates (such as ribose, lactulose, and glutaraldehyde) and organic acids (such as glutaric acid, methylmalonic acid, and oxalic acid) from our data also pointed to the aberrant energy metabolism at the occurrence of adenomyosis.

**Fig. 5:**
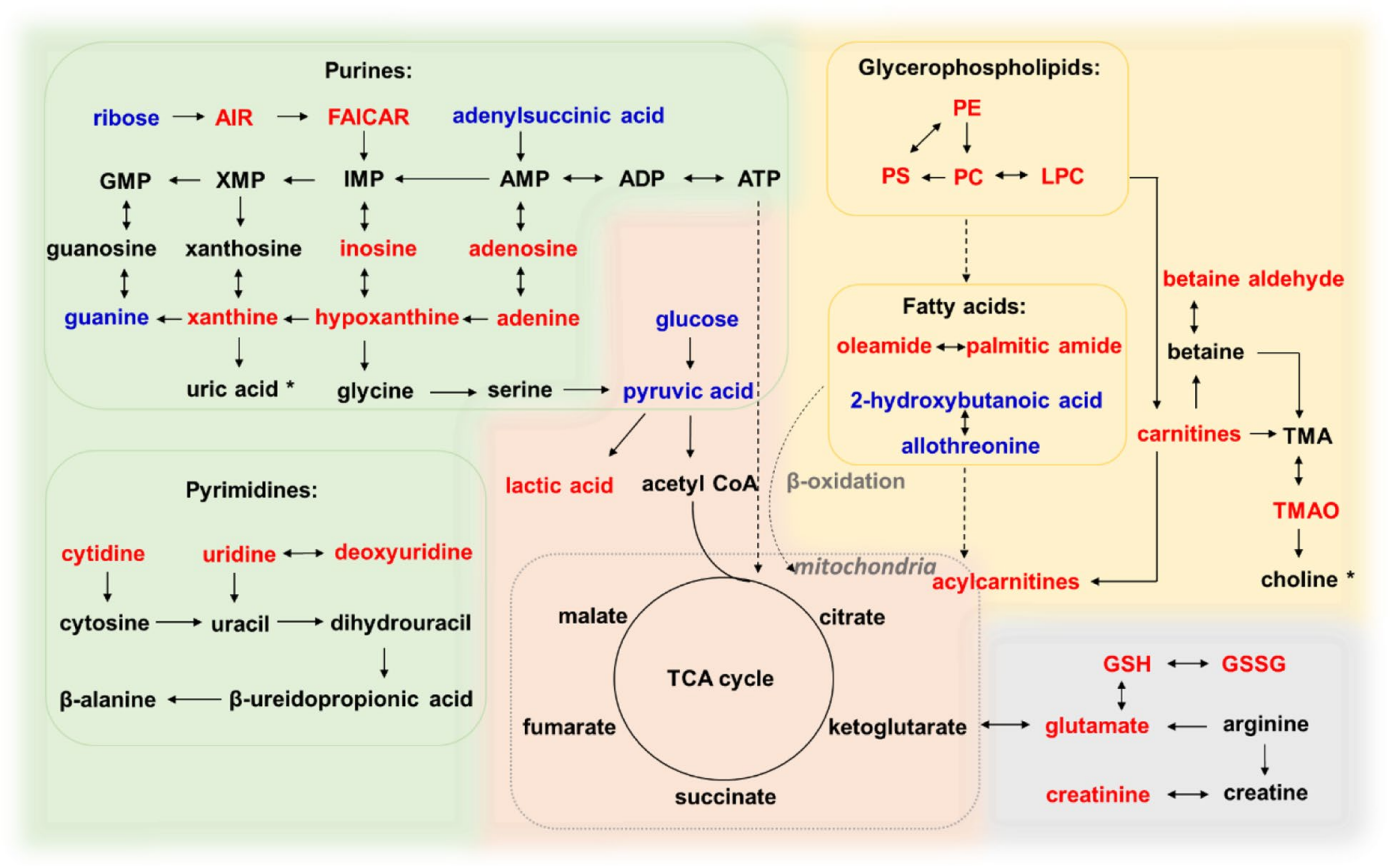
A putative model depicts the metabolic changes in myometrium of adenomyosis patients. Red text represents significantly elevated metabolites (e.g., inosine), and blue text represents significantly depleted metabolites (e.g., ribose). AIR, 5-aminoimidazole ribonucleotide; FAICAR, phosphoribosyl formamidocarboxamide; PE, phosphatidylethanolamine; PS, phosphatidylserine; PC, phosphatidylcholine; LPC, lysoPC; TMA, trimethylamine; TMAO, trimethylamine N-oxide; GSH, glutathione; GSSG, oxidized glutathione; TCA, tricarboxylic acid; *, metabolites significantly elevated in the serum of adenomyosis patients according to a previous report [12]

Last but not least, purine metabolism was particularly the most aberrant pathway in adenomyosis group compared to the controls (Table 2, Fig. 5). Purine nucleotides are principal constituents for cellular energy store (such as ATP) and participate directly in the regulation of DNA replication. Adenosine, a vital molecule of purinergic signalling, along with its downstream metabolites adenine, inosine, xanthine, and hypoxanthine, were significantly elevated in adenomyosis group. Regarding these abnormalities in purine metabolism, it could be related to increased inflammation and oxidative stress that involved in the pathophysiology of adenomyosis [46, 47]. In this study, pyrimidine metabolism followed the similar pattern to that of purines. The concentrations of some pyrimidines and their downstream metabolites (uridine and deoxyuridine) were also increased in adenomyosis group, which indicated enhanced function in nucleic acid metabolism.

To the best of our knowledge, this is the first study investigated the global metabolic changes in the myometrium of adenomyosis patients. The advantages of this research over previous studies lie in the novelty of the subject, as well as the integrative elucidation of the metabolic profiles. We analyzed the metabolomic signatures in tissue around the lesion to obtain an integrated biology map of this disease, which revealed the pathogenic pathways more directly. The aberrant metabolites and underlying pathways in adenomyosis cases could be mediated in the pathogenesis of adenomyosis. Thus they might be potential targets in treating adenomyosis. However, as for precise targets and how the therapies would arise, we realized that there was still a long way to go. Although there are significant molecular variations in the myometrium of adenomyosis patients, the identification of key mediators remains challenging. Further studies are required to determine the clinical significance of the metabolic aberrations.

Our study also has several limitations. This is a preliminary study using untargeted metabolomics approach, in which the metabolic markers still need further validation with larger sample size in the future. Additionally, the present research focused on the metabolic profiles of myometrium. It would be interesting to evaluate metabolic alternations in the endometrium. Furthermore, a serological test is still warrant. In our follow-up study we will explore the adenomyosis-associated features in the serum metabolome using the integrative metabolomics approach, while specific focus could be placed on the significant altered metabolites and pathways found in this study.

## Conclusion

In summary, we illustrated the global metabolome characteristics of the myometrium in adenomyosis patients for the first time. A total of 106 aberrant metabolites and the related metabolism pathways underlying adenomyosis development were picked out, in which oxidative stress, inflammation, cellar proliferation, and energy metabolism were mainly involved. These findings provide comprehensive insights into the intricate metabolic networks of adenomyosis, which also demonstrated the superiority of metabolomics in pathophysiology study. The results could be utilized as references for further clinical examination, as well as provide certain inspiration for the potential therapeutic targets of adenomyosis.

## Supplementary Information

**Additional file 1: Figure S1.** Representative photomicrographs of hematoxylin and eosin-stained uterine cross-sections from women with adenomyosis and without adenomyosis. **Figure S2.** Pathway analysis of significant altered metabolites. **Table S1.** Detailed information about the 106 significant changed metabolites.

## Data Availability

All data are included in this article and its additional files.
