# Supplementary material for: Integrative metabolomic profiling reveals aberrations in myometrium associated with adenomyosis: a pilot study
Source: Reprod Biol Endocrinol. 2022 Mar 9;20:49. doi: 10.1186/s12958-022-00914-5 (PMC8905769; doi:10.1186/s12958-022-00914-5)
Supplement: Supplementary file 1 — Additional file 1: Figure S1. Representative photomicrographs of hematoxylin and eosin-stained uterine cross-sections from women with adenomyosis and without adenomyosis. Figure S2. Pathway analysis of significant altered metabolites. Table S1. Detailed information about the 106 significant changed metabolites. [file 12958_2022_914_MOESM1_ESM.docx]

**Supplementary Information**

**Integrative metabolomic profiling reveals aberrations in myometrium associated with adenomyosis: a pilot study**

Wei Song^1^, Zhibo Zhang^2^, Ying Jiang^3^, Yang Cao^2^, Bo Zhang^1^, Yujie Wang^1^, Honghui Shi^2*^, Lan Zhu^2*^

1. Medical Science Research Center, State Key Laboratory of Complex Severe and Rare Diseases, Peking Union Medical College Hospital, Chinese Academy of Medical Science and Peking Union Medical College, 100730 Beijing, China

2. Departments of Obstetrics and Gynecology, Peking Union Medical College Hospital, Chinese Academy of Medical Sciences and Peking Union Medical College, National Clinical Research Center for Obstetric and Gynecologic Disease, 100730 Beijing, China.

3. Department of pathology, Peking Union Medical College Hospital, Chinese Academy of Medical Sciences and Peking Union Medical College, 100730 Beijing, China.

* Correspondence: Honghui Shi, honghuishi@sina.com; Lan Zhu, zhu_julie@sina.com

Figure S1. Representative photomicrographs of hematoxylin and eosin-stained myometrium cross-sections from uteri with adenomyosis (A) and without adenomyosis (B). The arrows pointed to the presence of ectopic endometrial tissue (endometrial glands and stroma) penetrating the myometrium.

Figure S2. Pathway analysis of significant altered metabolites

Table S1. Detailed information about the 106 significant changed metabolites

| **No.** | **metabolite** | **HMDB No.** | **class** | **sub-class** | **platform** | **rt (min)** | **m/z** | **mean-AM** | **mean-CON** | **VIP** | **P-value** | **Q-value** | **fold change** |
| --- | --- | --- | --- | --- | --- | --- | --- | --- | --- | --- | --- | --- | --- |
| 1 | xanthine | HMDB00292 | Nucleosides, nucleotides, and analogues | Purines and derivatives | GC | 1151.51 | 353 | 4.7392 | 1.2505 | 1.72 | 0.000066 | 0.005681 | 3.7898 |
| 2 | adenosine | HMDB00050 | Nucleosides, nucleotides, and analogues | Purines and derivatives | LC-positive | 168.95 | 268.1037 | 4.2245 | 2.0256 | 2.45 | 0.001512 | 0.026507 | 2.0855 |
| 3 | N6-methyladenosine | HMDB0004044 | Nucleosides, nucleotides, and analogues | Purines and derivatives | LC-positive | 134.11 | 282.1195 | 0.2202 | 0.1303 | 1.90 | 0.002387 | 0.033423 | 1.6903 |
| 4 | inosine | HMDB00195 | Nucleosides, nucleotides, and analogues | Purines and derivatives | GC | 1437.10 | 73 | 991.4161 | 698.1246 | 1.54 | 0.031675 | 0.124472 | 1.4201 |
| 5 | adenine | HMDB00034 | Nucleosides, nucleotides, and analogues | Purines and derivatives | LC-positive | 157.27 | 136.0613 | 18.2801 | 10.2275 | 1.98 | 0.000621 | 0.016709 | 1.7874 |
| 6 | hypoxanthine | HMDB0000157 | Nucleosides, nucleotides, and analogues | Purines and derivatives | LC-negative | 212.77 | 135.0300 | 0.1998 | 0.1400 | 2.25 | 0.020126 | 0.101353 | 1.4271 |
| 7 | 5'-methylthioadenosine | HMDB0001173 | Nucleosides, nucleotides, and analogues | Purines and derivatives | LC-positive | 97.26 | 298.0965 | 0.5550 | 0.2666 | 1.60 | 0.010210 | 0.072178 | 2.0814 |
| 8 | S-adenosylhomocysteine | HMDB0000939 | Nucleosides, nucleotides, and analogues | Purines and derivatives | LC-positive | 401.50 | 385.1274 | 0.6368 | 0.4521 | 1.48 | 0.002238 | 0.032338 | 1.4085 |
| 9 | guanine | HMDB0000132 | Nucleosides, nucleotides, and analogues | Purines and derivatives | LC-positive | 221.14 | 152.0565 | 10.4246 | 18.5631 | 2.19 | 0.000025 | 0.004010 | 0.5616 |
| 10 | adenylsuccinic acid | HMDB0000536 | Nucleosides, nucleotides, and analogues | Purines and derivatives | LC-negative | 304.89 | 457.2364 | 0.0015 | 0.0035 | 1.53 | 0.048600 | 0.149584 | 0.4339 |
| 11 | purine riboside | HMDB29956 | Nucleosides, nucleotides, and analogues | Purines and derivatives | GC | 1346.10 | 71 | 2.2927 | 3.1500 | 2.28 | 0.000132 | 0.007811 | 0.7279 |
| 12 | NAD | HMDB0000902 | Nucleosides, nucleotides, and analogues | Purines and derivatives | LC-negative | 426.48 | 662.0891 | 0.0037 | 0.0024 | 1.40 | 0.030997 | 0.123269 | 1.5282 |
| 13 | phosphoribosyl formamidocarboxamide | HMDB0001439 | Nucleosides, nucleotides, and analogues | Pyrimidine and derivatives | LC-positive | 212.99 | 367.0562 | 0.0965 | 0.0855 | 1.43 | 0.034359 | 0.128974 | 1.1278 |
| 14 | deoxyuridine | HMDB0000012 | Nucleosides, nucleotides, and analogues | Pyrimidine and derivatives | LC-negative | 316.89 | 227.0660 | 0.0055 | 0.0030 | 2.29 | 0.031050 | 0.123363 | 1.8487 |
| 15 | CDP-ethanolamine | HMDB0001564 | Nucleosides, nucleotides, and analogues | Pyrimidine and derivatives | LC-positive | 441.26 | 447.0676 | 0.0893 | 0.0594 | 1.44 | 0.017907 | 0.096041 | 1.5044 |
| 16 | citicoline | HMDB0001413 | Nucleosides, nucleotides, and analogues | Pyrimidine and derivatives | LC-negative | 436.90 | 487.0895 | 0.0099 | 0.0062 | 1.82 | 0.013397 | 0.083065 | 1.5864 |
| 17 | uridine | HMDB0000296 | Nucleosides, nucleotides, and analogues | Pyrimidine and derivatives | LC-positive | 160.93 | 245.0764 | 2.8225 | 2.2786 | 1.61 | 0.014863 | 0.087521 | 1.2387 |
| 18 | cytidine | HMDB0000089 | Nucleosides, nucleotides, and analogues | Pyrimidine and derivatives | LC-positive | 235.19 | 244.0924 | 0.3909 | 0.2035 | 2.49 | 0.002173 | 0.031869 | 1.9216 |
| 19 | cytidine monophosphate N-acetylneuraminic acid | HMDB0001176 | Nucleosides, nucleotides, and analogues | Pyrimidine and derivatives | LC-negative | 435.78 | 613.1277 | 0.0135 | 0.0097 | 1.91 | 0.007086 | 0.059806 | 1.3892 |
| 20 | DTDP-4-acetamido-4,6-dideoxy-galactose | HMDB0012222 | Nucleosides, nucleotides, and analogues | Pyrimidine and derivatives | LC-positive | 442.55 | 590.1212 | 0.9097 | 0.6169 | 2.48 | 0.003347 | 0.039884 | 1.4745 |
| 21 | cytidine 2'-phosphate | HMDB0011692 | Nucleosides, nucleotides, and analogues | Pyrimidine and derivatives | LC-positive | 439.22 | 324.0589 | 0.1740 | 0.1331 | 1.67 | 0.004270 | 0.045393 | 1.3073 |
| 22 | 5-aminoimidazole ribonucleotide | HMDB0001235 | Nucleosides, nucleotides, and analogues | Pyrimidine and derivatives | LC-positive | 23.49 | 296.0560 | 0.0250 | 0.0179 | 1.27 | 0.036681 | 0.132625 | 1.3992 |
| 23 | acetylcarnitine (C2) | HMDB0000201 | Lipids and lipid-like molecules | carnitines | LC-positive | 297.39 | 204.1233 | 167.2554 | 107.1339 | 1.65 | 0.018185 | 0.096744 | 1.5612 |
| 24 | propionylcarnitine (C3) | HMDB0000824 | Lipids and lipid-like molecules | carnitines | LC-positive | 272.19 | 218.1381 | 2.4948 | 1.2883 | 1.42 | 0.036327 | 0.132083 | 1.9366 |
| 25 | butyrylcarnitine (C4) | HMDB0002013 | Lipids and lipid-like molecules | carnitines | LC-positive | 249.38 | 232.1548 | 23.6762 | 10.5098 | 1.77 | 0.009415 | 0.069153 | 2.2528 |
| 26 | decanoylcarnitine (C10) | HMDB0000651 | Lipids and lipid-like molecules | carnitines | LC-positive | 182.89 | 316.2480 | 0.5641 | 0.2179 | 1.43 | 0.001797 | 0.028989 | 2.5888 |
| 27 | dodecanoylcarnitine (C12) | HMDB0002250 | Lipids and lipid-like molecules | carnitines | LC-positive | 175.20 | 344.2795 | 1.3986 | 0.4498 | 2.34 | 0.000058 | 0.005413 | 3.1097 |
| 28 | 5-tetradecenoylcarnitine (C14) | HMDB0002014 | Lipids and lipid-like molecules | carnitines | LC-positive | 169.54 | 370.2950 | 1.5840 | 0.5296 | 2.22 | 0.000196 | 0.009462 | 2.9912 |
| 29 | l-palmitoylcarnitine (C16) | HMDB0006461 | Lipids and lipid-like molecules | carnitines | LC-positive | 163.43 | 400.3424 | 33.4792 | 11.7234 | 2.19 | 0.000055 | 0.005295 | 2.8558 |
| 30 | hexadec-2-enoyl carnitine (C16:1) | HMDB0006317 | Lipids and lipid-like molecules | carnitines | LC-positive | 164.56 | 398.3264 | 6.7702 | 2.4540 | 2.15 | 0.000189 | 0.009318 | 2.7588 |
| 31 | heptadecanoyl carnitine (C17) | HMDB0006210 | Lipids and lipid-like molecules | carnitines | LC-positive | 161.92 | 414.3575 | 0.9363 | 0.3503 | 2.44 | 0.000001 | 0.001027 | 2.6730 |
| 32 | stearoylcarnitine (C18) | HMDB0000824 | Lipids and lipid-like molecules | carnitines | LC-positive | 159.86 | 428.3743 | 27.2030 | 7.4282 | 2.94 | 0.000002 | 0.001085 | 3.6621 |
| 33 | elaidic carnitine (C18:1) | HMDB0006464 | Lipids and lipid-like molecules | carnitines | LC-positive | 160.48 | 426.3591 | 49.3572 | 18.8582 | 1.98 | 0.000742 | 0.018160 | 2.6173 |
| 34 | linoelaidyl carnitine (C18:2) | HMDB0006461 | Lipids and lipid-like molecules | carnitines | LC-positive | 162.54 | 424.3427 | 15.5147 | 6.7120 | 1.69 | 0.003514 | 0.040868 | 2.3115 |
| 35 | oleamide | HMDB0002117 | Lipids and lipid-like molecules | Fatty acids and fatty alcohols | LC-positive | 34.27 | 282.2795 | 16.7354 | 6.2945 | 2.15 | 0.001186 | 0.023124 | 2.6587 |
| 36 | palmitic amide | HMDB0012273 | Lipids and lipid-like molecules | Fatty acids and fatty alcohols | LC-positive | 34.33 | 256.2632 | 1.7089 | 0.9365 | 1.71 | 0.007496 | 0.061511 | 1.8248 |
| 37 | 1-hexadecanol | HMDB03424 | Lipids and lipid-like molecules | Fatty acids and fatty alcohols | GC | 1119.35 | 71 | 4.0294 | 5.3791 | 2.08 | 0.002678 | 0.035440 | 0.7491 |
| 38 | dodecanol | HMDB11626 | Lipids and lipid-like molecules | Fatty acids and fatty alcohols | GC | 857.27 | 71 | 1.3704 | 1.8578 | 2.15 | 0.000302 | 0.011999 | 0.7377 |
| 39 | 2-hydroxybutanoic acid | HMDB00008 | Lipids and lipid-like molecules | Fatty acids and fatty alcohols | GC | 508.58 | 131 | 8.3914 | 17.8509 | 1.86 | 0.001637 | 0.027645 | 0.4701 |
| 40 | allothreonine | HMDB04041 | Lipids and lipid-like molecules | Fatty acids and fatty alcohols | GC | 725.37 | 281 | 0.1878 | 0.3666 | 1.23 | 0.008351 | 0.064927 | 0.5123 |
| 41 | lysopc(18:4) | HMDB0010389 | Lipids and lipid-like molecules | Glycerophospholipids | LC-positive | 358.43 | 516.3093 | 0.1850 | 0.0711 | 2.64 | 0.017355 | 0.094611 | 2.6032 |
| 42 | pc(15:0/16:1) | HMDB0007936 | Lipids and lipid-like molecules | Glycerophospholipids | LC-positive | 143.66 | 718.5404 | 1.3789 | 0.9309 | 1.30 | 0.046990 | 0.147308 | 1.4812 |
| 43 | pc(15:0/18:2) | HMDB0007940 | Lipids and lipid-like molecules | Glycerophospholipids | LC-positive | 142.20 | 744.5569 | 2.1130 | 1.4467 | 1.45 | 0.014202 | 0.085462 | 1.4606 |
| 44 | pc(18:1/15:0) | HMDB0008066 | Lipids and lipid-like molecules | Glycerophospholipids | LC-positive | 143.23 | 746.5731 | 1.6896 | 1.0864 | 1.42 | 0.009572 | 0.069748 | 1.5553 |
| 45 | pc(20:0/18:4) | HMDB0008273 | Lipids and lipid-like molecules | Glycerophospholipids | LC-positive | 122.29 | 810.6062 | 139.6689 | 101.7815 | 1.23 | 0.033866 | 0.128177 | 1.3722 |
| 46 | pc(20:3/15:0) | HMDB0008396 | Lipids and lipid-like molecules | Glycerophospholipids | LC-positive | 85.88 | 770.5698 | 2.6876 | 1.9877 | 1.18 | 0.030504 | 0.122374 | 1.3521 |
| 47 | ps(14:0/20:0) | HMDB0112295 | Lipids and lipid-like molecules | Glycerophospholipids | LC-positive | 79.63 | 764.5263 | 2.1814 | 1.6192 | 1.10 | 0.005216 | 0.050620 | 1.3472 |
| 48 | ps(15:0/18:0) | HMDB0112322 | Lipids and lipid-like molecules | Glycerophospholipids | LC-positive | 60.65 | 750.5465 | 25.5470 | 17.4189 | 1.06 | 0.016903 | 0.093404 | 1.4666 |
| 49 | ps(18:4/22:2) | HMDB0112505 | Lipids and lipid-like molecules | Glycerophospholipids | LC-positive | 41.72 | 836.5473 | 2.1957 | 1.4817 | 1.43 | 0.000380 | 0.013453 | 1.4819 |
| 50 | pe(16:0/20:4) | HMDB0008937 | Lipids and lipid-like molecules | Glycerophospholipids | LC-positive | 110.60 | 740.5207 | 3.0924 | 2.1382 | 2.29 | 0.000069 | 0.005759 | 1.4462 |
| 51 | pe(20:3/16:1) | HMDB0009353 | Lipids and lipid-like molecules | Glycerophospholipids | LC-positive | 88.20 | 740.5257 | 3.6284 | 2.5959 | 1.72 | 0.004966 | 0.049177 | 1.3978 |
| 52 | 5-demissine | HMDB0033029 | Lipids and lipid-like molecules | Steroids | LC-positive | 364.50 | 1016.5456 | 0.0467 | 0.0323 | 1.29 | 0.042513 | 0.141195 | 1.4471 |
| 53 | 2-methoxyestrone 3-glucuronide | HMDB0004482 | Lipids and lipid-like molecules | Steroids | LC-positive | 378.72 | 477.2124 | 0.1946 | 0.1556 | 1.21 | 0.045775 | 0.145718 | 1.2503 |
| 54 | androsterone sulfate | HMDB0002759 | Lipids and lipid-like molecules | Steroids | LC-negative | 29.11 | 369.1629 | 0.3261 | 0.5691 | 1.57 | 0.039019 | 0.136096 | 0.5731 |
| 55 | dehydroepiandrosterone sulfate | HMDB0001032 | Lipids and lipid-like molecules | Steroids | LC-negative | 28.13 | 367.1468 | 0.6261 | 0.9335 | 1.79 | 0.036686 | 0.132633 | 0.6708 |
| 56 | chaconine | HMDB0039353 | Lipids and lipid-like molecules | Steroids | LC-positive | 301.96 | 852.5204 | 0.0468 | 0.0954 | 2.44 | 0.007774 | 0.062613 | 0.4905 |
| 57 | glutamic acid | HMDB00148 | Amino acids and derivatives | - | GC | 886.60 | 188 | 3.3662 | 2.2185 | 1.58 | 0.015007 | 0.087959 | 1.5174 |
| 58 | gamma-glutamylalanine | HMDB0006248 | Amino acids and derivatives | - | LC-positive | 291.18 | 219.0969 | 0.5223 | 0.2259 | 1.65 | 0.007225 | 0.060396 | 2.3115 |
| 59 | N-acetylaspartylglutamic acid | HMDB0001067 | Amino acids and derivatives | - | LC-negative | 441.22 | 303.0812 | 0.0141 | 0.0073 | 1.99 | 0.045969 | 0.145975 | 1.9316 |
| 60 | valine | HMDB0000883 | Amino acids and derivatives | - | LC-positive | 25.24 | 118.0890 | 0.0314 | 0.0268 | 1.56 | 0.012564 | 0.080431 | 1.1690 |
| 61 | kynurenine | HMDB0000684 | Amino acids and derivatives | - | LC-positive | 256.95 | 209.0914 | 0.0938 | 0.0459 | 2.07 | 0.002554 | 0.034574 | 2.0444 |
| 62 | saccharopine | HMDB0000279 | Amino acids and derivatives | - | LC-positive | 25.24 | 277.1381 | 0.0396 | 0.0321 | 1.99 | 0.000601 | 0.016463 | 1.2321 |
| 63 | isoleucyl-leucine | HMDB0028911 | Amino acids and derivatives | - | LC-positive | 182.72 | 245.1854 | 0.2039 | 0.0823 | 1.02 | 0.007417 | 0.061189 | 2.4769 |
| 64 | leucyl-serine | HMDB0028938 | Amino acids and derivatives | - | LC-positive | 272.60 | 219.1410 | 0.3114 | 0.1587 | 1.37 | 0.035334 | 0.130529 | 1.9620 |
| 65 | valyl-isoleucine | HMDB0029130 | Amino acids and derivatives | - | LC-positive | 196.61 | 231.1698 | 0.2390 | 0.1486 | 1.43 | 0.036546 | 0.132419 | 1.6086 |
| 66 | alanyl-lysine | HMDB0060442 | Amino acids and derivatives | - | LC-positive | 430.27 | 218.1493 | 0.2858 | 0.2269 | 1.48 | 0.011605 | 0.077187 | 1.2597 |
| 67 | glutathione | HMDB00125 | Amino acids and derivatives | - | GC | 1235.41 | 213 | 11.5143 | 3.9628 | 1.35 | 0.000180 | 0.009119 | 2.9056 |
| 68 | oxidized glutathione | HMDB0003337 | Amino acids and derivatives | - | LC-positive | 486.85 | 613.1622 | 3.2993 | 2.1540 | 2.42 | 0.000432 | 0.014258 | 1.5317 |
| 69 | citrulline | HMDB0000904 | Amino acids and derivatives | - | LC-positive | 382.70 | 176.1021 | 0.1896 | 0.2326 | 1.43 | 0.036372 | 0.132152 | 0.8150 |
| 70 | phenylalanine | HMDB0000159 | Amino acids and derivatives | - | GC | 905.86 | 218 | 68.2490 | 102.2568 | 1.71 | 0.029797 | 0.121064 | 0.6674 |
| 71 | histidinyl-serine | HMDB0028894 | Amino acids and derivatives | - | LC-positive | 361.93 | 243.1083 | 0.0220 | 0.0600 | 2.05 | 0.002697 | 0.035574 | 0.3659 |
| 72 | hydroxyprolyl-valine | HMDB0028876 | Amino acids and derivatives | - | LC-positive | 412.40 | 231.1389 | 0.0053 | 0.0167 | 2.03 | 0.000095 | 0.006586 | 0.3181 |
| 73 | N-acetyl-l-leucine | HMDB11756 | Amino acids and derivatives | - | GC | 753.49 | 57 | 0.7803 | 1.3380 | 1.74 | 0.033551 | 0.127661 | 0.5832 |
| 74 | glutaric acid | HMDB00661 | Organic acids and derivatives | - | GC | 741.08 | 57 | 4.0227 | 6.8888 | 1.73 | 0.013255 | 0.082629 | 0.5839 |
| 75 | methylmalonic acid | HMDB00202 | Organic acids and derivatives | - | GC | 576.66 | 281 | 1.2122 | 1.9951 | 1.63 | 0.021490 | 0.104463 | 0.6076 |
| 76 | oxalic acid | HMDB02329 | Organic acids and derivatives | - | GC | 512.62 | 147 | 6.1466 | 13.3095 | 2.26 | 0.000626 | 0.016764 | 0.4618 |
| 77 | pyruvic acid | HMDB00243 | Organic acids and derivatives | - | GC | 443.54 | 174 | 9.4214 | 14.2961 | 1.21 | 0.021703 | 0.104955 | 0.6590 |
| 78 | maleamate | HMDB0000176 | Organic acids and derivatives | - | GC | 771.82 | 73 | 2.6705 | 4.0700 | 1.78 | 0.028885 | 0.119324 | 0.6561 |
| 79 | O-phosphoethanolamine | HMDB0000224 | Organic acids and derivatives | - | LC-negative | 479.47 | 140.0098 | 0.0886 | 0.0625 | 2.00 | 0.044314 | 0.143739 | 1.4166 |
| 80 | glycerylphosphorylethanolamine | HMDB0000114 | Organic acids and derivatives | - | LC-positive | 385.46 | 216.0626 | 0.6105 | 0.3602 | 2.03 | 0.005203 | 0.050548 | 1.6949 |
| 81 | 2-hydroxyethanesulfonate | HMDB0003903 | Organic acids and derivatives | - | LC-negative | 160.19 | 124.9894 | 0.0277 | 0.0201 | 1.80 | 0.001146 | 0.022720 | 1.3774 |
| 82 | creatinine | HMDB0000562 | Organic acids and derivatives | - | LC-positive | 337.66 | 114.0649 | 2.3304 | 2.0186 | 1.68 | 0.016288 | 0.091708 | 1.1544 |
| 83 | lactic acid | HMDB0001311 | Organic acids and derivatives | - | LC-negative | 226.32 | 89.0242 | 2.0482 | 1.5212 | 2.17 | 0.001650 | 0.027763 | 1.3464 |
| 84 | taurine | HMDB00251 | Organic acids and derivatives | - | GC | 935.15 | 174 | 379.0166 | 265.2207 | 1.44 | 0.004779 | 0.048099 | 1.4291 |
| 85 | N-ornithyl-taurine | HMDB0033519 | Organic acids and derivatives | - | LC-positive | 211.71 | 240.1012 | 0.2930 | 0.2027 | 1.29 | 0.027276 | 0.116182 | 1.4453 |
| 86 | N-acetyl-glucosamine-phosphate | HMDB0001367 | Carbohydrates | - | LC-negative | 446.97 | 300.0467 | 0.0687 | 0.0427 | 2.95 | 0.001268 | 0.024003 | 1.6069 |
| 87 | gluconic acid | HMDB00625 | Carbohydrates | - | GC | 1102.61 | 244 | 19.5829 | 12.4260 | 1.31 | 0.027943 | 0.117464 | 1.5760 |
| 88 | ribose-5-phosphate | HMDB01548 | Carbohydrates | - | GC | 1199.66 | 315 | 1.4936 | 0.8213 | 1.29 | 0.041194 | 0.139254 | 1.8187 |
| 89 | dihydroxyacetone | HMDB01882 | Carbohydrates | - | GC | 616.03 | 57 | 4.4242 | 8.5250 | 1.20 | 0.035636 | 0.131007 | 0.5190 |
| 90 | glucose | HMDB00122 | Carbohydrates | - | GC | 1080.09 | 73 | 1823.4220 | 3219.9798 | 2.55 | 0.000216 | 0.010015 | 0.5663 |
| 91 | glutaraldehyde | HMDB29599 | Carbohydrates | - | GC | 647.77 | 71 | 2.0236 | 2.6242 | 1.21 | 0.045910 | 0.145897 | 0.7712 |
| 92 | lactulose | HMDB00740 | Carbohydrates | - | GC | 1483.65 | 267 | 0.1925 | 0.2663 | 1.99 | 0.008991 | 0.067526 | 0.7232 |
| 93 | ribose | HMDB00283 | Carbohydrates | - | GC | 937.20 | 307 | 0.8017 | 1.3504 | 2.03 | 0.032498 | 0.125896 | 0.5937 |
| 94 | guanidinopropionic acid | HMDB0013222 | Organic nitrogen compounds | - | LC-negative | 337.13 | 130.0610 | 0.0232 | 0.0147 | 1.11 | 0.039907 | 0.137381 | 1.5818 |
| 95 | trimethylamine N-oxide | HMDB0000925 | Organic nitrogen compounds | - | LC-positive | 322.52 | 76.0745 | 0.1227 | 0.0760 | 1.34 | 0.027125 | 0.115914 | 1.6139 |
| 96 | betaine aldehyde | HMDB0001252 | Organic nitrogen compounds | - | LC-positive | 344.95 | 102.0901 | 0.7256 | 0.5620 | 1.54 | 0.047617 | 0.148134 | 1.2911 |
| 97 | N-lactoyl ethanolamine | HMDB0032356 | Organic nitrogen compounds | - | LC-positive | 337.67 | 134.0799 | 0.8714 | 0.7519 | 1.57 | 0.018619 | 0.097820 | 1.1589 |
| 98 | 2-hydroxypyridine | HMDB13751 | Organoheterocyclic compounds | - | GC | 436.30 | 152 | 100.0689 | 186.2820 | 2.11 | 0.000692 | 0.017529 | 0.5372 |
| 99 | quinolinic acid | HMDB00232 | Organoheterocyclic compounds | - | LC-negative | 352.22 | 166.0176 | 0.0050 | 0.0110 | 1.20 | 0.022848 | 0.107502 | 0.4551 |
| 100 | 5,6,7,8-tetrahydro-4-methylquinoline | HMDB0029709 | Organoheterocyclic compounds | - | LC-positive | 34.52 | 148.1126 | 0.0598 | 0.1692 | 1.48 | 0.001497 | 0.026361 | 0.3537 |
| 101 | riboflavin | HMDB0000244 | Organoheterocyclic compounds | - | LC-positive | 208.27 | 377.1453 | 0.2411 | 0.1886 | 1.48 | 0.008704 | 0.066385 | 1.2784 |
| 102 | pyrrole-2-carboxylic acid | HMDB04230 | Organoheterocyclic compounds | - | LC-negative | 477.38 | 110.0235 | 0.0047 | 0.0026 | 2.11 | 0.000221 | 0.010164 | 1.8197 |
| 103 | thiamine monophosphate | HMDB0002666 | Organoheterocyclic compounds | - | LC-positive | 414.93 | 345.0842 | 0.3281 | 0.1830 | 2.70 | 0.000104 | 0.006864 | 1.7924 |
| 104 | equol | HMDB0002209 | others | Phenylpropanoids | LC-negative | 336.41 | 241.0903 | 0.0030 | 0.0016 | 1.31 | 0.012808 | 0.081220 | 1.8660 |
| 105 | catechol | HMDB00957 | others | Benzenoids | GC | 668.89 | 69 | 2.1734 | 3.1452 | 1.95 | 0.006277 | 0.056141 | 0.6910 |
| 106 | toluenesulfonic acid | HMDB59933 | others | Benzenoids | GC | 907.21 | 229 | 3.7345 | 5.0037 | 1.34 | 0.043009 | 0.141909 | 0.7464 |

Notes: AM, adenomyosis group; CON, control group.
